# “I love being a midwife; it's who I am”: A Glaserian Grounded Theory Study of why midwives stay in midwifery

**DOI:** 10.1111/jocn.15078

**Published:** 2019-11-10

**Authors:** Dianne Bloxsome, Sara Bayes, Deborah Ireson

**Affiliations:** ^1^ Edith Cowan University Joondalup WA Australia; ^2^ Edith Cowan University Bunbury WA Australia

**Keywords:** job satisfaction, midwifery, qualitative, workforce

## Abstract

**Aims and objectives:**

To understand why Western Australian (WA) midwives choose to remain in the profession.

**Background:**

Midwifery shortages and the inability to retain midwives in the midwifery profession is a global problem. The need for effective midwifery staff retention strategies to be implemented is therefore urgent, as is the need for evidence to inform those strategies.

**Design:**

Glaserian grounded theory (GT) methodology was used with constant comparative analysis.

**Methods:**

Fourteen midwives currently working clinically area were interviewed about why they remain in the profession. The GT process of constant comparative analysis resulted in an overarching core category emerging. The study is reported in accordance with Tong and associates’ (2007) Consolidated Criteria for Reporting Qualitative Research (COREQ).

**Results:**

The core category derived from the data was labelled—“I love being a midwife; it's who I am.” The three major categories that underpin the core category are labelled as follows: “The people I work with make all the difference”; “I want to be ‘with woman’ so I can make a difference”; and “I feel a responsibility to pass on my skills, knowledge and wisdom to the next generation.”

**Conclusion:**

It emerged from the data that midwives’ ability to be “with woman” and the difference they feel they make to them, the people they work with and the opportunity to “grow” the next generation together underpin a compelling new middle‐range theory of the phenomenon of interest.

**Relevance to clinical practice:**

The theory that emerged and the insights it provides will be of interest to healthcare leaders, who may wish to use it to help develop midwifery workforce policy and practice, and by extension to optimise midwives’ job satisfaction, and facilitate the retention of midwives both locally and across Australia.

**What does this paper contribute to the wider global clinical community?**

- The inability to retain midwives in the profession is a global issue; the number of studies investigating midwives job satisfaction is low in number.
- The findings from this study add a valuable contribution to the body of work on midwifery workforce retention by providing an account of the factors that keep midwives’ in the profession.
- Identification of the reasons why midwives stay in midwifery is imperative to the sustainability and longevity of the profession.

## 1 INTRODUCTION

The inability to retain midwives in the midwifery profession is both an international and local problem (Adegoke, Atiyaye, Abubakar, Auta, & Aboda, 2015; Papoutsis, Labiris, & Niakas, 2014). This issue was highlighted in 2006 by the World Health Organization (WHO), and efforts were employed to improve the retention of midwives. Regardless of endeavours to improve the situation, the global retention of midwives remains of great concern (UNFPA, 2014; WHO, 2006). The WHO (2006) declared that midwives are the foundation to the decrease in maternal mortality, and has anticipated that in the event that the workforce maintenance issue is not tended to, at that point increments in maternal and neonatal mortality will follow. In 2011, the United Nations Population Fund (UNFPA, 2011) identified that there was an estimated global shortage of 350,000 midwives and too implemented change (UNFPA, 2011). In 2014, the UNFPA reported that despite extensive worldwide efforts the issue still exists and in some areas is worsening (UNFPA, 2014).

According to the Australian Government (2017), there were 26,369 midwives employed and working in the midwifery sector in 2017. This represents a decrease of 5.1% midwives since 2014, with an average annual decrease of 1.5%. The average age of the Australian midwife is currently 48 years. The full‐time equivalent (FTE) number of midwives is also decreasing across Australia, with an increasing number of midwives preferring to work part time. The combined factors of the ageing and depleting midwifery workforce plus a reduction overall in those working full time are of concern. The need for effective midwifery staff retention strategies to be implemented is therefore urgent, as is the need for evidence to inform those strategies (WHO, 2019).

The number of studies investigating midwives’ job satisfaction is low in number; however, the research that has previously been undertaken describes and explains the drivers of midwifery workforce attrition to include lack of recognition, stress, exhaustion, high workplace demands, rosters, on call, lack of management support, lack of family and social life, self‐neglect, staff shortages, lack of autonomy, inadequate budget, inadequate education and training, medical dominance, policy and protocols and wages. Collectively, these factors are all reportedly causing midwives to leave the profession (Curtis, Ball, & Kirkham, 2006; Geraghty, Speelman, & Bayes, 2018; Lavender & Chapple, 2004; Papoutsis et al., 2014; Wakelin & Skinner, 2007).

## 2 BACKGROUND

A recent integrative review undertaken by Bloxsome, Ireson, Doleman, and Bayes (2018) highlights a dearth in the research as to why midwives stay in the profession, with only two studies (Sullivan, Lock, & Homer, 2011; Watson, Potter, & Donohue, 1999) focusing on Australia. Neither provides an in‐depth account of the phenomenon. Further, there has been no work on this topic in Western Australia (WA), which arguably has additional workforce retention challenges due to its isolated geographical location. WA is the largest state in Australia with a total land area at over 2.5 million km^2^ and a population of over 2.5 million people (Australian Bureau of Statistics, 2019); this large vast space and large population leads to intrastate distance, and isolation of healthcare professionals in rural areas. The distance between WA and other states in Australia is between 2,000–4,000 kms (Google Maps, 2016) leading to interstate isolation. In 2014, 34,687 women gave birth in WA, which represents an increase of 2.2% from 2013 and is reported as being the highest annual number since 1974 when reporting began. A large number of these births (78.5%) were in the metropolitan health region and the remainder in country regions. The number of tertiary, public, private and homebirths was 16.5%, 41.6%, 40.5% and 0.6%, respectively (Government of Western Australia, 2018). WA has the second highest number of private hospitals in Australia, which anecdotally may help explain the high number of caesarean sections (34.9% in 2014); the rate has tripled over the past 22 years and is the second highest in the country (Government of Western Australia, 2018). Due to these unique geographical and population characteristics, findings from other contexts about why midwives stay or leave the profession are unlikely to apply in full to WA.

## 3 AIMS

The aim of this study was to understand why WA midwives chose to remain in the profession and was conducted for the purpose of understanding the factors leading to midwives remaining in their jobs.

The broad research question guiding this study was as follows: Why do WA midwives stay in midwifery?

## 4 METHODS

### 4.1 Study design

This study was undertaken using Glaserian grounded theory methodology (Glaser & Strauss, 1967). Grounded theory (GT) has become a widely used approach that can be employed in both qualitative and quantitative studies due to its flexibility (Roberts, 2008). GT can be defined as “seeking to identify and explain what is happening in a social setting” (Roberts, 2008, p. 679). GT uses an inductive process to generate a theory that can then be generalised and applied. The emergent theories are then said to be “grounded” as the theory emerges from the data (Schneider & Whitehead, 2013). Due to its nonprescriptive and objective approach, GT was deemed the most suitable methodology to answer the research question. By employing the steps in GT, a middle‐range theory that both describe and explain the phenomenon of interest emerged from the findings.

The study is reported following the Consolidated Criteria for Reporting Qualitative Research (COREQ) checklist as advocated by Tong, Sainsbury, and Craig (2007) (see File S1).

### 4.2 Study setting

The setting for this study was public and private metropolitan, rural and community midwifery practice sites within WA.

### 4.3 Participants

At the time this study was undertaken, there were just over 2,000 midwives registered in WA (Nursing & Midwifery Board of Australia, 2018a). In keeping with GT methodology, recruitment, data collection and data analysis occurred as a cyclical/iterative process (Glaser & Strauss, 1967). Three forms of sampling were used to recruit participants from this population. First, purposive sampling, also known as nonprobability sampling, was employed (Creswell, Hanson, Clark Plano, & Morales, 2007; Schneider & Whitehead, 2013). Currently practicing midwives with a minimum of 12 months of experience were invited via an electronic bulletin posted on the Facebook page of the authors’ employing organisation (which a number of midwives are known to “like” and “follow,” and which is available to view by anyone including those who are not Facebook users), to participate in the study. Second, the snowball technique was used. The snowball technique asks individuals who meet the study inclusion criteria to assist in recruiting others (Schneider & Whitehead, 2013). This approach was employed to ensure sample heterogeneity through maximum variation, and to ensure the theory generated from this study represented all midwifery practice contexts within WA, and occurred in this study through midwives who saw the Facebook post sharing it with their wider professional networks either on Facebook or by email. As data analysis progressed and emergent categories were saturated (after six interviews with midwives), theoretical sampling was employed (using the same process as was used initially) as the third recruitment strategy. Strauss and Corbin (1990) discuss theoretical sampling as a process of sampling on the basis of emerging concepts that can contribute to building the opening and axial coding of the theory. Theoretical sampling was used to “thicken” the data categories and generate a substantive theory of the factors that contribute to why WA midwives stay in the profession.

The sample for investigation involved midwives who had been practising for a minimum of 12 months and who were working in clinical midwifery environments in WA. A total of 23 midwives were provided with an information sheet via email, 16 verbally consented to participate, and 14 provided written consent and proceeded to be interviewed on one occasion each.

### 4.4 Data collection

Data were collected from December 2017–November 2018. Interview duration ranged from 60–120 min (average length of 60 min) with a total of 18.5 hr of interview data obtained. Data were listened to and transcribed verbatim by the first author, and to maintain participants’ anonymity in the reporting of direct quotes to support the narrative, codes were given to each participant, for example MW1 (which denotes the first participant midwife to be interviewed).

Semi‐structured, open‐ended in‐depth interviews were used to gather data for this study. This approach involved asking participants an initial set of open‐ended questions (see Table 1) that became more refined and guided by the interviewer as the participants’ responses emerged. Knox and Burkard (2009) support the use of this approach for qualitative studies, as to the participant it may seem more like a friendly conversation than a data‐gathering interview.

**Table 1:** Initial open‐ended questions

1. Can you please tell me how many years you have been a midwife for?
2. Can you please tell me what service you work in?
3. Can you please tell me what training you undertook to become a midwife?
4. Can you please now tell me why you stay in midwifery?

Interviews were conducted by the first author either face to face, via Skype™, or over the phone depending on the participant's geographical location and choice. All interviews were audio‐recorded with participants’ permission and transcribed verbatim. In keeping with grounded theory methodology, memos and field notes were taken during and after each interview.

### 4.5 Data analysis

Data analysis occurred simultaneously with data collection using the constant comparison approach consistent with the tenets of GT methodology (Glaser & Strauss, 1967). Each interview was transcribed verbatim and “first level” coded using Microsoft Word™. This meant data were analysed line‐by‐line and each incident relevant to the research question coded with a keyword or phrase. These codes were then compared to each other and alike codes grouped; codes were also considered against all emerging categories to ensure correct “fit” and with one another (Strauss & Corbin, 1990). Analysis of each interview occurred within one week of it occurring. As new data were obtained, the codes from them were compared to the previously developed categories, and this continued until no new information was heard (after six interviews) (see Table 2). At this point, theoretical saturation was reached (Strauss & Corbin, 1990). Theoretical sampling was then employed to ensure heterogeneity and to confirm and “thicken” the emergent theory; this occurred after 14 interviews.

**Table 2:** An example of the original expressions in the interviews, their substantive codes and categories

Original Quote	Substantive codes	Categories
“I go to work each day because I love the people that I work with… It's a huge reason why I stay” (MW14). “feel[ing] well supported within the team.. they are very supportive and encouraging” (MW2).	The people I work with make all the difference Having a supportive team is crucial	The people I work with make all of the difference
“it was my calling; once you do it you can't undo it and not consider yourself a midwife…If I had to describe myself it would be ‘Mama Midwife’” (MW10). “when I had my children I decided that I couldn't actually see myself doing anything else. It wasn't originally my calling but then it became it” (MW9).	I stay because midwifery is my calling I stay because midwifery is my calling	It's who I am
“…coaching women through her contractions can be tiring, but it's so satisfying” (MW11). “it is about being ‘with woman’”(MW1).	I want to be “with woman” so I can make a difference Being “with woman”	I want to be “with woman” so I can make a difference

### 4.6 Ethical considerations

Ethical approval for this study was granted by Edith Cowan University Human Research Ethics Committee (record no. 18,747) on 23 November 2017.

### 4.7 Data management

All participants were assured of confidentiality and anonymity and were provided with letters of introduction, information sheets and the opportunity to ask questions before consenting to participate, plus an explanation of the proposed research. Participants were assured that their involvement in the research was voluntary and that they were free to withdraw from the project at any time.

All raw data were stored in password‐protected computer files, along with transcribed interview data. Memos were stored in a locked filing cabinet. The research and data produced were managed in accordance with Human Research Ethics Committee guidelines and in accordance with National Health and Medical Research Council [NHMRC] requirements (NHMRC, 2013).

### 4.8 Trustworthiness

This study was overseen by an experienced grounded theorist who was involved in every step of the research to ensure rigour of the process. To ensure trustworthiness in this study, a number of measures were employed. A bracketing exercise was undertaken prior to the commencement of data collection to ensure prior knowledge and assumptions were put to one side in order to remain open‐minded (Husserl & Boyce, 1931). In keeping with grounded theory methodology, memos and field notes were taken during and after each interview (Glaser & Strauss, 1967). These memos and field notes formed part of the audit trail, whilst data analysis was occurring. Member checking also occurred with participants. This involved contacting participants for clarification of findings to ensure accuracy (Creswell et al., 2007). A further formal face‐to‐face member checking group was conducted with members of the research team and midwives from a range of WA practice contexts who had not participated in the study to confirm the trustworthiness of the analysis. The group was presented with the analysis of the data and the developed categories, and feedback was sought in relation to the credibility, authenticity and representativeness of the emergent theory. During the formal member checking group, all participants unanimously agreed that the findings and the final theory resonated strongly with them and were likely to represent why midwives stay in the profession.

## 5 RESULTS

### 5.1 Participant characteristics

The sample of 14 participants comprised eight midwives practising in rural locations in WA and six working in the greater Perth metropolitan area (see Table 3).

**Table 3:** Participants Demographics

Demographic variable	Category	Frequency
Years as a midwife	1–5	3
	10–20	8
	20–40+	3
Education	Masters of Midwifery Practice	2
	Post‐graduate Diploma of Midwifery	5
	Bachelor degree	3
	Hospital based midwifery program	4
Health service type ‐ Rural	Public Hospital	4
	Private Hospital	1
	Midwifery Group Practice	3
Health service type ‐ Metropolitan	Public Hospital	3
	Private Hospital	2
	Midwifery Group Practice	1
Gender	Female	14
	Male	0

**Figure 1:**
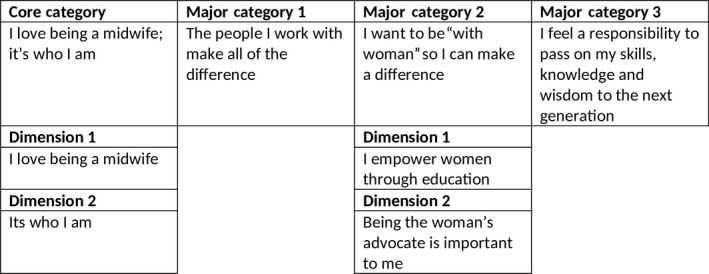
Core Category and Major Categories

The core category derived from the data was labelled—“I love being a midwife; it's who I am,” and comprised three contributory major categories that together represent why WA midwives stay in midwifery. The three major categories that underpin the core category are labelled as follows: “The people I work with make all the difference”; “I want to be ‘with woman’ so I can make a difference”; and “I feel a responsibility to pass on my skills, knowledge and wisdom to the next generation.” The core category and one major category are two‐dimensional (Figure 1).

### 5.2 Core category: I love being a midwife; it's who I am

It was overwhelmingly clear throughout the interview process that participants loved midwifery, were highly committed to the profession and felt that once one was a midwife, one was always a midwife: that it was in your heart and even if you left for a short period you would always return. The core category that represents why WA midwives stay in midwifery has two dimensions to it: “I love being a midwife” and “It's who I am.”

#### 5.2.1 Dimension 1: I love being a midwife

Participants unanimously agreed they stay in midwifery because they love it. Although participants were unable to define “love” when asked, it was a word they used repeatedly. Participants said for example, that they *“… love the job” (MW2), that they “..definitely stay for the love” (MW4),* they *“… stay because I love it” (MW6)* and that they *“… love being a midwife” (MW7 and MW9).* MW11 went further: she *“… fell in love with it (midwifery) and there was no thought of going back, I just loved it.*

Regardless of the midwifery environment each participant was working in, they all described this love for the profession and all that it entails. The participant midwives loved, for instance, the amazing experiences they described to occur on a day‐to‐day basis, the connection with women and the creation of a calm environment in which to welcome new life. Their passion for midwifery was evident in all interviews. MW2 captured it thus: “*I think its amazing being with women at that crucial part of their life‐giving birth is exciting and amazing and I love all those things about it.”* MW12 said she loves “*…that moment when the baby's born, it's just pure magic.”* MW12 went on to say that after years in midwifery, *“I still get(s) goose bumps each [birth].”* MW11 perhaps captured all participants’ overarching feeling about midwifery when she said *“It's the best job in the world as far as I'm concerned; there is nothing to touch it.”*

The participants reported taking pride in their work and being proud to be part of the midwifery profession and culture. MW8 explained, *“I am proud to be a midwife,”* MW10 similarly said:

“I'm proud that I’ve been a midwife for 17 years, I love it when one of my kid’s friends says to them I love your mum because she delivered me… or I was the first one to touch their head. It’s just really special…It’s a sense of pride, I'm really proud that I'm representing the women of [local area]… It’s just part of me I can’t let go of. I'm really proud that I'm a midwife, it’s my chosen path and I’ll stick with it until I retire.”

Participants recognised that midwifery was more than just a job to them and felt very lucky to be involved in such a special time in a childbearing woman's life. A large number of participants described their passion for the job as more than the money they earned, with some midwives alluding to the fact they would work for free if they did not have bills to pay. MW10 stated, “*I would not mind working for free at all; I’d love to go overseas and volunteer. I’d love to do it for free if life didn't depend on money.”* MW14 similarly quoted, “*I'm not in it for the money…money is a bonus for doing what I love. Whole heartedly I believe it's more than a job for me.”*

It was acknowledged by a large number of participants that money was in fact a bonus for them. MW3 said that she felt *“the money's an added bonus… because I do what I love.”* MW5 echoes *“it's just a bonus, it's a bonus for doing what I love,” and* MW11 captured all participants’ overarching feeling when she described midwifery as a hobby: “*It's almost like I think I'm getting paid to do this, this is fantastic. I really enjoy it and I'm actually getting paid to do it. It's like I'm getting paid to do my hobby, that's what it's like.”*

#### 5.2.2 Dimension 2: It's who I am

Midwives reported midwifery to be a large component of their lives; they also said it could not be “undone.” Being a midwife was who they were as a person, a mother, a wife, a friend and a daughter, with participants referring to themselves becoming and being a midwife as their “calling.” One participant said, *“it was my calling; once you do it you can't undo it and not consider yourself a midwife…If I had to describe myself it would be ‘Mama Midwife’” (MW10)*. MW1, MW3 and MW13 also reported midwifery to be their calling and not just a job*.*

One participant felt she was born into midwifery and she just could not let go of the profession. She reported “*… it just feels like we are born into [midwifery]… It's just part of me I can't let go of. I'm really proud that I'm a midwife. It's my chosen path and I’ll stick with it until I retire” (MW10).* Another participant reported midwifery being ingrained into her whilst growing up, *“it really did influence me what was normal in New Zealand and what was normal for my mum to talk about.. (it) had a really big impact on me. I think a lot of that's ingrained, my mother's attitude to birth which was my grandmother's and my great grandmother's” (MW5).*

Not all midwives felt they were born or called to be a midwife, with some midwives realising much later in life, after an event or “light bulb” moment, as happened for MW13: “*I was in a newsagent and I looked at a magazine that had mothers and babies on it and I just knew then that I had to be a midwife” (MW13).* For other participants, their “light bulb” moment was being involved in or witnessing a birth. MW14 reported “*I knew I wanted to be a nurse, and when I witnessed a birth in my training, I thought this was amazing and knew I wanted to do it (midwifery).”* Another participant felt that her “light bulb” moment was watching her sister throughout her pregnancy journey:

“My sister had her first baby when she was 17…seeing the changes that she went through as a person and watching this little human being that never existed before, come into the world, and change so much within the first few months, and the bond that she had, that was brought about by breastfeeding and just being there, it just kicked something off in me, and I thought ‘that’s what I want to do.” (MW3)

Their own experience of giving birth inspired others to become midwives. MW5 felt that “*it wasn't until I had my own my daughter, I don't know what clicked, I just thought I really want to do this (midwifery).”* Similarly, MW9 reported *“when I had my children I decided that I couldn't actually see myself doing anything else. It wasn't originally my calling but then it became it.”*

Participants very much enjoyed that being a midwife was more than just a job and they reported they felt that “it *certainly infiltrates to all aspects of my life” (MW7).* Participants also reported that midwifery is very much just who they are and that they had to remain with women to feel “authentic.” MW1 felt that:

“I worry that if I actually stop doing it I won’t be as authentic…I still don’t want to leave clinical practice because it’s still so much of who I am…I don’t want to lose sight of what it means to be with a woman at 2 o’clock in the morning as she’s birthing her baby because that’s affects how you teach and for me it will affect how I teach, it will affect research, it will affect everything.”

Participants also commented they felt that, “once *a midwife always a midwife” (MW14)*. MW11 similarly said that she also felt that, *“I think once you've been a midwife you've always got that thing inside of you.”*

### 5.3 Major category 1: The people I work with make all of the difference

The role of a midwife has changed over the years, but essentially it is to provide women with high‐quality, evidence‐based care throughout their pregnancy, childbirth and postnatal period. The data from this study suggest that midwives cannot provide holistic support to women, however, without collegial support in the working environment. As one participant stated, “*having such a supportive team is crucial to working in this model (continuity of midwifery care); if I didn't have colleagues who got it and could support me I couldn't do it… it has to be a reciprocal caring for each other” (MW1).*

All participants interviewed agreed that the people they worked with played a large role in why they stayed in the profession. MW14 reported that “*I go to work each day because I love the people that I work with… It's a huge reason why I stay.”* These people ranged from the other midwives to doctors and support staff. MW3, for example, said “*the majority (of midwives) make it worthwhile and that's another reason why I choose to stay.”* The team environment and camaraderie this created seemingly enabled midwives to go to work each day. MW3 stated *“Camaraderie keeps you going”*; MW14 similarly said “*the people I work with is a huge reason as to why I stay.. It's the colleagues around you, you have support coming from everywhere… it's the camaraderie of the team.”*

One participant described the collegial support she was afforded as a safeguard and felt that because her colleagues “had her back” she would be less likely to make a mistake:

“I know the midwives I work with at home, we all have each other’s back and we support each other. I know the chances of me making a mistake at home are much less because I’ve got somebody who will be with me, we watch each other and we safe guard each other and we safe guard the woman together and it’s a lovely way of working, and that second midwife doesn’t go until the first midwife is happy that everything is ok.” (MW1)

The overwhelming support participants felt from the people they work with was voiced strongly throughout the interviews, with MW2 reporting *“feel[ing] well supported within the team.. they are very supportive and encouraging,” and* MW5 felt *“that support is always there.”* MW6 too explained that her *“…colleagues 100% supported me.”* Further participants reporting the same with MW8 *stating “I’ve never been left unsupported.. You need to have supportive colleagues,”* MW11 found that *“within the labour ward.. It's very supportive.”* One participant (MW12) described how this support extended to being cared for personally as well as in the professional environment: she reported having had to take time off work for medical reasons and that her colleagues went *“out of their way*” to help her during this time.

Having a good team environment was also reported as making a difference to participants as evidence by MW11:

“Labour ward work is intense and terrifying at times but you build this incredible bond with this team you work with because you’ve been through some really horrible times together, some really sad times and then you just pull it all together. That team work for me is just phenomenal.”

Another participant reported that she had *“…a really good team down here (rural hospital). We have a great rapport and trust within the team” (MW12).* MW7 similarly said “*we are like a family up here we fall in and out of love with each other… but ultimately, we work well. I’d be stacking shelves at Woollies [a supermarket] if I didn't have that relationship.”*

“Respect” was another factor that was reportedly important to participants and denoted another reason as to why they choose to stay in the profession. MW6 reports having “mutual respect.. I feel like all of us were always in a huddle,” “I’m well respected… I’ve been working in the field for a long time; the respect goes both ways – between the doctors and the midwives” (MW4). Participants also reported having a good working relationship with their colleagues; this relationship was built of mutual respect, like‐mindedness and support. One participant felt that the doctors really had “her back”: “we have two gorgeous Drs here [private rural facility]… It’s the support, it’s never wavering, it’s always there, they don’t mind if you have questions. Its lovely to know they’ve (the Drs) always got my back and support me” (MW10). MW13 reports “I am very keen on teamwork… I have to have a good relationship with my team when ‘the rubber hits the road’ so they will do what I ask.”

Participants reported the team they work with is like a second family to them. This “family” support network made all the difference and made it enjoyable to go to work each day and was a significant reason cited by all participants as to why they stay in midwifery. The following statements clearly demonstrate this.

“To work with people that are so committed to what they do and who obviously also enjoy what they do and we’ve developed real nice friendships, so that’s what keeps me going. They’ve become like my second family which is great, it’s like they are my sisters and cousins which is lovely.” (MW11)

“My team picked my kids up and took them home when I was at a birth (big smiles)… one of us in the morning always rings and says I’m coming in shall I bring coffee? … for the three weeks that my husband is away one of the husbands has offered to pick up my kids from school (big smiles)… we just look after each other.” (MW5)

“When I'm in labour and birth suite it’s not uncommon for my partner or one of my partners’ (midwife) husbands to bring me in breakfast so that I can be sustained…I am on several of my work colleagues school pickup lists, I help and bath the kids, feed them and put them to bed while my work colleague is at a birth. But that’s what you need to do to be sustainable. That is an element of our job because otherwise it just doesn’t work.” (MW7)

### 5.4 Major category 2: I want to be “with woman” so I can make a difference

This second major category comprises two dimensions. The first is labelled “I empower women through education” and the second labelled “Advocating for women is important to me.”

Midwives being “with woman” is the central tenet to providing woman‐centred midwifery care in partnership with women; it is embedded in midwifery philosophy as well as the Australian midwife standards of practice (Nursing & Midwifery Board of Australia, 2018b). The relationship midwives have with women was a priority for the participants interviewed. Being “with woman" were words that featured strongly throughout interviews with participants and the concept was spoken of with high regard. MW1 states *“it is about being ‘with woman’,”* and MW2 and MW3 echoed the same view that they enjoyed being “with woman” and felt that midwifery was in fact a profession that was aimed at being “with woman.”

Participants described that they enjoyed being “with woman” because they felt they could make a significant difference to women in their care. This difference could be in the antenatal period, during labour and birth and/or helping the woman make the transition to motherhood. The difference participants said they could make to women represented a strong reason for choosing to stay in the midwifery profession. MW11 described the difference she feels that she makes:

“It’s being with women and watching women have the ability to actually make their dreams, their plans come true. To watch them in labour and then watch them produce these babies, it never loses its magic for me – ever. Despite all the other crap that goes on at times, that’s what really makes it.”

Participants felt they made a difference to women and their families and wanted to make their childbearing journey better for them in any way they could. Being able to facilitate this gave participants job satisfaction and subsequently led to them remaining in the profession. MW9 believes “*the care I provide makes a difference, we have low epidural rates, we have built up a relationship with the woman, she wants to birth in her community, [and] we have built that trust. We have time to be with them.”* Another participant similarly stated:

“It’s not about catching babies for me, it’s about seeing that amazing transformation of a woman and a man becoming parents (big smile), I love it, I just love it, that look of look what we just produced and it doesn’t matter if it’s a Caesar(ean) or a vaginal birth, it’s just that creating of a family, I think that’s so important to nurture that and give them the confidence that’s it’s not about… – I mean I can’t remember the last time I delivered a baby, probably last Christmas I reckon, that doesn’t worry me. So, just seeing that and supporting that in any way I can.” (MW14)

Participants reported feeling satisfied when helping women to become mothers and the transition to parenthood. MW9 stated *“they have a baby [and] you are helping them be new parents.”* MW11 felt that “…*coaching women through her contractions can be tiring, but it's so satisfying.”* MW12 enjoyed “*…helping the mums to look after their babies over those first couple of days, just to help ease them through those first couple of days as much as you can.”*

Being able to share in such an intimate moment was described as such a joyful experience to be involved in. As MW10 said “*we are invited into such a beautiful, intimate moment in life, it's so special. Those women have the trust and faith an invite us into that room, that moment to share that pregnancy journey. It's very special to be there.”* MW2 similarly said, “*I think it's amazing being with women at that crucial part of their life ‐ giving birth is exciting and amazing and I love all those things about it.”*

When participants spoke about the difference they feel they make they beamed with pride, and in some participants’ stories, the difference they recalled having made was so considerable that tears welled in their eyes as they told their stories. The selfless dedication to women heard in the participants’ narratives was truly remarkable and was clear evidence of truly loving their job.

#### 5.4.1 Dimension 1: I empower women through education

The midwife plays a significant role in educating women. The aim of the midwife in educating women is to empower women and enable them to make informed choices throughout their childbearing journey. When women feel educated and supported, they can make decisions that are right for them, which in turn lead to positive birth experiences. A large number of participants reported that providing education to women was extremely important to them and thus facilitated the empowerment of women in their care. Participants stated, *“giving them (women) that choice” (MW3);* “*Doing the job I do you're in a good position to do that (educate), its education and information, and that's the power to make those changes that work for you and your baby. I like that side of it…empowerment through education” (MW4); and I have a fire in my belly for antenatal and having choice…it's about empowering women” (MW8).*

The antenatal period is an opportunity to provide women with education. The information provided to women in this period can significantly impact their birth experience. Participants felt very strongly about this and reported it as an important component of their job. The participants conveyed taking a great deal of pride in empowering women, which led to an increased job satisfaction, subsequently leading to them stay in the profession. MW1 said, “*I really have come to believe that the midwife makes a big difference, but if I can do my job right antenatally … (it) doesn't matter who is with (the woman) on the day (she gives birth because) she knows she can do it.”* Another participant reiterate this: “*I’ve been teaching antenatal classes since I was a midwife, I'm so passion about giving them the right information and the right education and ironing out those*…* wives tales, there's so many of them out there… it's just exciting” (MW14)*.

The transition to motherhood and the creation of the family unit is another important aspect in the midwife's role and was reported throughout a number of the interviews. MW13, for example, said *“there's nothing better than teaching them (women) how to do it themselves, I do that with dads as well. Teaching, teaching, teaching, I might spend an hour with them. That's where I do get a thrill setting her up for success.”* MW14 stated “*Empowering the woman postnatally is so important… I need to know that they (women) are well educated*.”

#### 5.4.2 Dimension 2: Being the woman's advocate is important to me

Midwives advocate on a daily basis for women in their care to ensure the provision of safe, women‐centred midwifery care. The midwives in the study are staunch advocates for women and take this component of their role very seriously. Participants interviewed felt that being the woman's advocate was important to them and reported this as another reason why they stay in midwifery. Participants reported such sentiments as *“being a woman's advocate (that is) often lacking in other models… that's the reason why I stay” (MW1);* “*being the woman's advocate is very important to me and that the woman gets what she wants, that's my philosophy” (MW2); and “they (women) know that someone is going to listen to them, believe them and stick up for them. Trying to navigate themselves through our system is a minefield, the language we use, having local knowledge, demystifying the whole process for them” (MW7).*

Participants reported feeling they needed to “fight the fight” for women in order for women to birth the way that they wanted. MW1 declared “*I will always fight for my women; even when I know I’m fighting a losing battle I will still fight it.”* Another participant said that she *“had lots of friends that have not had the birth they want, I get annoyed when people say she's got healthy baby, what more does she want, actually it's not just about that. You need to be there for her and facilitate plans, her birth, her labour so that her and her partner get what they want” (MW11).*

One participant referred to herself as *“being like a dog with a bone,”* when it comes to advocating for women, particularly when the woman's wishes were at odds with medical opinion, and expressing that surely her arguments would have an impact if she has “the discussion” enough times with the doctor.

“My managers have come to recognise that with me and they kind of roll their eyes and I say, you know I have to do this, you know I have to have this discussion with you, you know I have to take this to a doctor and I know that last week for another woman they said no but this is this woman, and I have to do it. I guess part of me is like a dog with a bone in thinking eventually surely it’ll have an impact.” (MW1)

Some participants referred to themselves as a “gate keeper” and described feeling the need to keep doctors out of the labour and birth room to enable women to have the birth they want: *“Many times, I have put my foot on the door and stopped the doctor from getting in”* (MW11); *“I'm definitely the gate keeper… when you talk about the advocation role, its huge… I'm comfortable to challenge them (the obstetrician) now and that's because I now have the confidence to” (MW14)*.

### 5.5 Major category 3: I feel a responsibility to pass on my skills, knowledge and wisdom to the next generation

**Figure 2:**
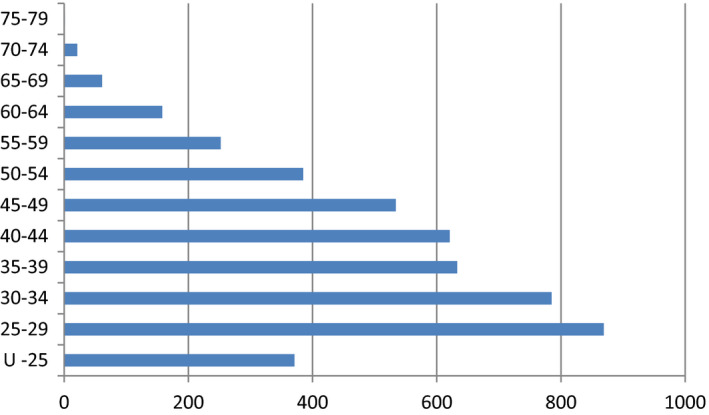
Midwife registration by age group. Data sourced from: Nursing and Midwifery Board of Australia Registrant Data (2017) [Colour figure can be viewed at http://www.wileyonlinelibrary.com]

As stated above, the average age of the Australian midwife is 48 years, with a large number of midwives facing retirement in the next 10–15 years (see Figure 2). It was therefore not surprising to hear that participants, many of whom were in this age bracket, were forward‐thinking and reported a drive to pass on their skills, knowledge and wisdom to the next generation as another reason for staying in the profession.

To feel able to retire, the older participant midwives described needing to know that the next generation is going to be able to continue to stay strong and resist the medical dominance. One participant said she felt she had to *“keep the fight going and teach students that are our next generation” (MW1*). Midwife participants felt strongly about the need to stay in the profession so they could help and support not only student midwives but also junior midwives to protect and promote midwifery. “*We have to continue to fight the fight for the women otherwise the medical model will just take over… I need to keep the younger ones (midwives) strong enough to resist the changes” (MW11).*

Further responses from midwives revealed they would “pass on the baton” and “hang up their Pinard's” when the next wave was ready to support women. “*I am at that part of my career now that, not to pass the baton on but to get the next wave there, ready to step up” (MW6).* “*I turn 50 next year and I will probably will give up MGP (midwifery group practice) at 55 (years old) and hang up my Pinard's and let the next generation of midwives through” (MW7).*

Sharing in their passion for midwifery and nurturing the next generation was also another important aspect as to why participants stayed in the profession; MW14 reports, *“If I can share passion of midwifery with student nurses and get more into the profession… my mission is to get them thinking about midwifery and to join the best profession in the world.”* MW7 similarly echoes this view: *“I just see it as a human right and justice; we are nurturing the new generation coming through. This is an important job.”*

## 6 DISCUSSION

This study essentially highlights that midwives’ ability to employ what intrinsically motivates them leads directly to job satisfaction and the intention to stay in their jobs and in the profession. A number of theories and models have been used to explain the phenomenon of job satisfaction, for example Maslow's hierarchy of needs theory (Maslow & Lewis, 1987), Herzberg's motivator‐hygiene theory (Herzberg, 1968) and Hackman’s (1980) job characteristics model.

In a previous study relating to midwives’ intention to stay (Papoutsis et al., 2014), the findings were related to Herzberg's six factors for job satisfaction, namely “achievement,” “recognition,” “the work itself,” “responsibility,” “advancement” and “growth” (Herzberg, 1968). All but one of these factors, however, represents extrinsic organisational drivers, whereas for our participants, only the job itself, and working in a team that was supportive and respectful and having the opportunity to develop and share skills, knowledge and abilities were the key considerations.

Deci and Ryan’s (1985) self‐determination theory (SDT), which provides a broad framework by which to explain human motivation and personality, helps explain the findings of this study. SDT posits three needs that are essential to maintain or enhance intrinsic motivation: the need for competence, relatedness and autonomy (Deci & Ryan, 2000). Competence refers to feeling a sense of accomplishment in activities and being capable of overcoming challenges. Relatedness reflects the need to have meaningful relations with significant others and a sense of belongingness and autonomy relates to the ability to have choice and not be controlled by others (Deci & Ryan, 2000).

The core category derived from our data, “I love being a midwife; it's who I am,” and represents that participants found their jobs both motivating and satisfying. It was clearly evident in the data that the title “midwife” is one participant took seriously, that they love their job and that they love being “with woman” when they are at their most vulnerable. McAra‐Couper et al. (2014) reported participants in their study also expressed “that midwifery is ‘more than a job’; a midwife is someone they become; and a way of life” (p. 31). La Guardia (2009) discusses fulfilling the need for competence relies on “doing,” or the engagement of activities that a person finds satisfying.

Being a midwife is seemingly part of one's identity. Being a midwife permeates into all aspects of one's life, it is who they are, and for some participants, it is what they have always wanted to be. La Guardia (2009) explores the concept of “who am I?” relating to identity and self; the importance of identity arising from the innate environment seemingly fulfils one's true or authentic potential. When a person's values and identity come together, this results in self‐determined motivation (Deci, Cascio, & Krusell, 1975).

Professional identity has been well researched in the areas of nursing (Browne, Wall, Batt, & Bennett, 2018; MacIntosh, 2003) and education (Beijaard, Meijer, & Verloop, 2004; Losano, Fiorentini, & Villarreal, 2018), but there is paucity focusing solely on midwives. The term professional identity addresses an individual's affiliation and attraction to a profession and their sense of belonging. Professional identity is made through one's beliefs and attitudes, values, motives and experiences that people align themselves in their current or anticipated career (Trede, Macklin, & Bridges, 2012). Hunter and Warren (2013) describe professional identity as integrated with personal identity. Hunter and Warren report that when midwives feel a sense of professional belonging and professional identity this contributed to resilience.

The joy being a midwife brings was reported by participants and reflects findings in other studies (Kirkham, Morgan, & Davies, 2006; Sullivan et al., 2011; Versaevel, 2011). A person engages in intrinsically motivated activities for the satisfaction and enjoyment it brings (Deci et al., 1975). The commitment midwives felt to the women in their care clearly represents their dedication to their values and beliefs and the identity achievement evidenced when this commitment is made (La Guardia, 2009).

Participants in this study felt intrinsically motivated when they were “with woman” and could see the difference their care made. The findings in this study reflect those found in other studies (Kirkham et al., 2006; McAra‐Couper et al., 2014; Papoutsis et al., 2014). The sheer enjoyment this engendered to the midwives in our study was an important factor in meeting their individual fulfilment needs (Deci et al., 1975) and by extension their job satisfaction.

Second to this, the major category, labelled *“the people I work with make all the difference*,” demonstrated the need for participants to feel they had meaningful significant relationships with others. This too was identified as an important reason for staying in the profession. Similarly, McAra‐Couper et al. (2014) identified in their study that midwives spoke of the midwifery community as whole, and these relationships assisted in the sustainability of practice. This is also seen in other studies (Kirkham et al., 2006; Lavender & Chapple, 2004; Papoutsis et al., 2014; Sandall, 1997; Sullivan et al., 2011) who all report the people they work with led to an increased job satisfaction and staff morale. Ryan and La Guardia (2000) enumerate that if relatedness is fulfilled, intrinsic motivation will result.

Essentially, women need midwives who are happy in their profession, the relationship that midwives have with their colleagues plays a crucial role in their ability to remain in their jobs. When midwives feel supported and encouraged, they can make and stand by their clinical decisions that can make a difference to the quality of the woman's childbearing experience. The relationship that participants have with their colleagues plays a crucial role in their ability to remain in the midwifery profession. The respect, support and family‐like work environment, all contribute to the participant's ability to be sustainable as a midwife.

There is a strong linkage between autonomy and competence (Nix, Ryan, Manly, & Deci, 1999). Nix and colleagues postulate that if a person is autonomously motivated, they will feel a positive state of vitality resulting in enhanced energy. If the needs for autonomy and competence are allowed, intrinsic motivation will result (Deci & Ryan, 2000) as well as factors of resilience (Vansteenkiste & Ryan, 2013). Autonomy generally relates to an individual, in these findings it was seen to relate to the woman in the midwife's care, as well as to the midwives themselves.

Autonomy is an important aspect in the sustainability of the midwifery profession. When individuals are able to autonomously regulate their own behaviour even when under pressure, their ability to cope with their environment is enhanced (Deci, Olafsen, & Ryan, 2017). This was also evident in Hunter and Warren's study who found that a strong sense of autonomy was central to resilience (Hunter & Warren, 2013). This is not a new finding in midwifery retention studies with a number of papers alluding to the same (Curtis et al., 2006; Kirkham et al., 2006; McAra‐Couper et al., 2014; Sullivan et al., 2011). The ability to practice autonomously increases midwives resilience due to the sense of independence felt; the satisfaction midwives feel when they are able to use their knowledge increases their “sense of usefulness” (Sabzevari & Rad, 2019). The degree of autonomy that midwives can practice is strongly influenced by the hierarchy of power due to the medical ideology in which midwives in WA are forced to practice. Although midwives report their frustration and find it challenging working within this medical ideology, they have a realistic expectation of what they can control and optimise their scope of practice accordingly (Kruger & McCann, 2018).

To date, there are no known Australian or international research studies undertaken on the use of SDT in the midwifery profession. SDT has, however, been employed in other areas as a way to increase job satisfaction. Arshadi (2010) supports the use of SDT with autonomy support in the workplace as a way to increase job satisfaction and intrinsic motivational needs and ultimately work motivation and job performance. We found this to be the case in our study, in that our findings suggest that if basic psychological needs are met this leads to positive work outcomes and increase job satisfaction.

## 7 CONCLUSIONS

The findings from this study add a valuable contribution to the body of work on midwifery workforce retention by providing an account of the factors that keep midwives in the profession. It emerged from the data that midwives’ ability to be “with woman” and the difference they feel they make to them, the people they work with and the opportunity to “grow” the next generation together underpin a compelling new middle‐range theory of the phenomenon of interest.

There are a number of limitations to the study, the first of which is that the data represent one geographical location and may not be generalisable across other midwifery contexts in Australia and internationally. Second, no male midwives, new graduate midwives, or Aboriginal or Torres Strait Islander midwives were interviewed; therefore, it cannot be assumed that these subgroups’ reasons for staying in the profession are the same as for those who participated, and further research is needed to understand what keeps these midwives in midwifery. These limitations notwithstanding the findings provide valuable previously unknown insights into what drives retention in this profession.

The correlation between job dissatisfaction and employee turnover is evident in the literature and is indisputable. To deflect the impending midwife shortage, it is vital that workplace policies focus on the maximisation of midwives’ job needs fulfilment. The support to practice autonomously clearly enhances job satisfaction. Reference to motivation strategies would be helpful, alongside the wider adoption of continuity of midwifery models that increase midwives’ autonomy and enable them to be “with woman.” The provision of time to effectively mentor less‐experienced midwives is also potentially crucial to workforce retention. Those involved in preregistration midwifery education may wish to consider the findings from our study in the employability and career development aspects of their courses. System change may also ensue as a result of imbuing midwifery students with knowledge about what keeps midwives in midwifery, as new graduates may then demonstrate a preference for jobs in workplaces that demonstrably support those professional needs.

## 8 RELEVANCE TO CLINICAL PRACTICE

The findings from this research have uncovered the reasons as to why midwives stay in the profession in one state in Australia. The theory that emerged and the insights it provides will be of interest to healthcare leaders. Similarly, the insight provided by the findings will help develop midwifery workforce policy and practice, and by extension to optimise midwives’ job satisfaction, and facilitate the retention of midwives both locally and across Australia.

## CONFLICT OF INTEREST

“No conflict of interest has been declared by the authors.”
